# Structure, activation and dysregulation of fibroblast growth factor receptor kinases: perspectives for clinical targeting

**DOI:** 10.1042/BST20180004

**Published:** 2018-12-13

**Authors:** Brendan Farrell, Alexander L. Breeze

**Affiliations:** Astbury Centre for Structural Molecular Biology, Faculty of Biological Sciences, University of Leeds, Leeds LS2 9JT, U.K.

**Keywords:** drug discovery and design, fibroblast growth factor receptors, receptor tyrosine kinases, structural biology

## Abstract

The receptor tyrosine kinase family of fibroblast growth factor receptors (FGFRs) play crucial roles in embryonic development, metabolism, tissue homeostasis and wound repair via stimulation of intracellular signalling cascades. As a consequence of FGFRs’ influence on cell growth, proliferation and differentiation, FGFR signalling is frequently dysregulated in a host of human cancers, variously by means of overexpression, somatic point mutations and gene fusion events. Dysregulation of FGFRs is also the underlying cause of many developmental dysplasias such as hypochondroplasia and achondroplasia. Accordingly, FGFRs are attractive pharmaceutical targets, and multiple clinical trials are in progress for the treatment of various FGFR aberrations. To effectively target dysregulated receptors, a structural and mechanistic understanding of FGFR activation and regulation is required. Here, we review some of the key research findings from the last couple of decades and summarise the strategies being explored for therapeutic intervention.

## Introduction

**Figure 1.**
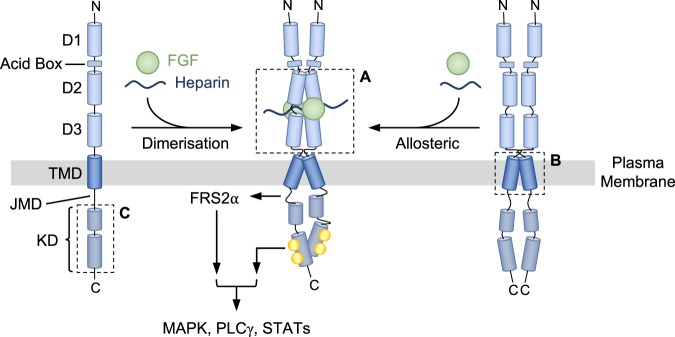
Stimulation of FGFRs. FGFRs are composed of an extracellular domain comprising D1, acid box, and D2 and D3 domains, followed by a single helix TMD, the JMD, and an intracellular ‘split’ tyrosine KD. Two models describing receptor stimulation by FGF ligand and heparin/heparan sulfate cofactor have been described: the canonical ligand-induced receptor dimerisation model (left) and an allosteric ligand-induced conformational change model (right). Receptor activation leads to *trans*-autophosphorylation of the kinase domains and stimulation of intracellular signalling cascades. The boxed regions (A–C) correspond to those in Figure 2.

Through their role in signal transduction pathways, protein kinases mediate a plethora of cellular phenotypic changes such as cell growth, proliferation, differentiation and survival [1]. Receptor tyrosine kinases (RTKs) are an important kinase subfamily whose members span the cell surface and activate intracellular signalling cascades in response to exogenous growth signals via binding of family-specific extracellular ligands. Canonically, this is achieved through ligand-driven receptor dimerisation and subsequent *trans*-autophosphorylation of the cytosolic receptor tyrosine kinase domains, stimulating kinase activity [2]. Alternatively, in cases where receptors are believed to exist as constitutive dimers, activation can be achieved allosterically via ligand-induced conformational rearrangements of the receptors. Fibroblast growth factor receptors (FGFRs), the focus of this review, are one of these RTK subfamilies, responding to the binding of fibroblast growth factors (FGFs) (Figure 1) [2,3]. Through their activation, FGFRs have roles in embryonic development, tissue homeostasis and metabolism [4–7].

The FGFR family is composed of four separately encoded yet highly homologous receptors, FGFRs 1–4, sharing between 56 and 71% sequence identity [8]. Structurally, all members share the same architecture consisting of a large ligand-binding extracellular domain (ECD) that comprises three immunoglobulin (Ig)-like domains (D1–3); a single membrane-spanning helix; and an intracellular domain containing the catalytically active ‘split’ tyrosine kinase domain (Figure 1). Genetic, biochemical and structural studies have yielded extensive insights into understanding of FGFR activation.

## Localisation of FGF ligand binding and receptor dimerisation

**Figure 2.**
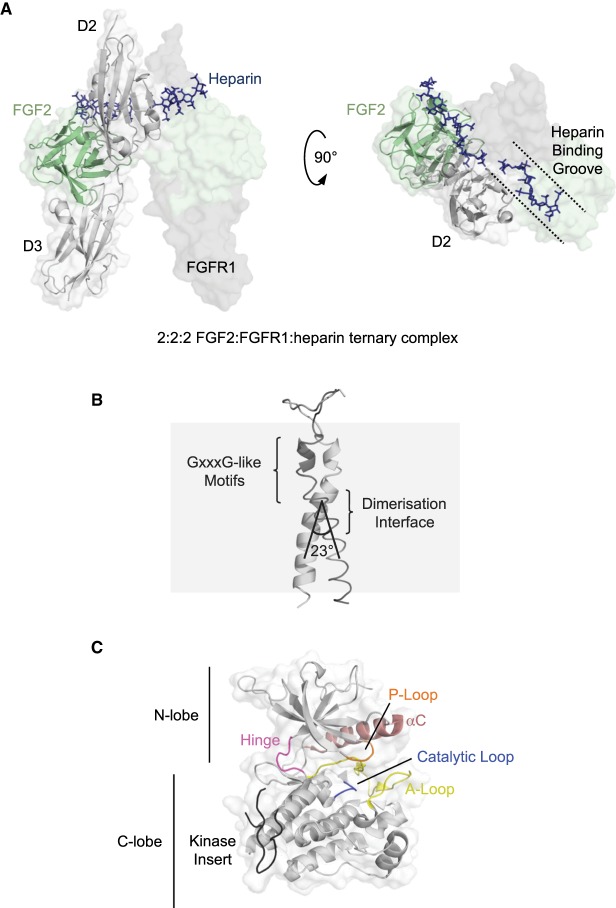
Structures of FGFR extracellular, transmembrane, and kinase domains. (**A**) Crystal structure of FGFR1 extracellular domains D2 and D3 (grey cartoon on transparent surface representation) in a 2:2:2 complex with FGF2 (light green, cartoon) and heparin (dark blue, sticks) (PDB: 1FQ9). Only one copy of FGF2 and FGFR1 are shown in cartoon representation for clarity. (**B**) An FGFR3 transmembrane domain dimer derived from NMR (PDB: 2LZL) in cartoon representation with the observed dimerisation interface and GxxxG-like motifs highlighted. (**C**) FGFR3 kinase domain crystal structure (PDB: 4K33) in cartoon representation on a transparent surface with the N- and C-lobes and structural elements, the αC helix (salmon), the P-loop (orange), the catalytic loop (blue), the A-loop (yellow), the kinase hinge (magenta) and the (incomplete) kinase insert (black) highlighted. Panels are not in scale with one another.

In mammals, there are 18 FGF ligands which can be subdivided into paracrine and endocrine families; all FGFs have a beta-trefoil fold with a heparan sulfate binding-site on its surface that facilitates sequestration of FGF ligands close to the cell surface for receptor binding [9,10]. FGF-ligand binding to FGFRs is localised to domains D2 and D3 of the extracellular domain [11], and in the case of paracrine FGFs, occurs in association with heparan sulfate proteoglycan cofactors [10] (Figure 1). While FGFs are able to bind independently to FGFRs in a 1:1 stoichiometry [12–14], heparan sulfate (or heparin) is necessary for receptor dimerisation and FGFR signalling [15–17]. Although dimerisation of a variety of 1:1 FGF:FGFR extracellular domain complexes was initially observed crystallographically in the absence of heparin [13,14,18], this dimerisation is likely to be a crystallisation artefact. However, these heparin-free crystallographically dimerised complexes were nonetheless useful for building models of heparin-mediated receptor dimerisation and are structurally similar to a 2:2:2 stoichiometry FGF2:FGFR1:heparin ternary complex structure solved at a later date (Figure 2) [19]. The stoichiometry and minimal heparan sulfate chain length required for receptor dimerisation have been disputed, resulting in different proposed models of FGFR activation [19–23]. However, regardless of the model, in all FGF:FGFR complex structures, FGF ligands make contacts with residues from D2, the D2–D3 linker and D3 domains of FGFRs, with the interfaces characterised by both hydrophobic and polar interactions. In the 2:2:2 stoichiometry ternary complex model, both FGF ligands and heparin make contacts with both FGFR molecules of the dimer (Figure 2). Unlike paracrine FGFs, endocrine FGFs such as FGF21 and FGF23 exhibit lower binding affinity for heparan sulfate [10] and require Klotho co-receptors to act as cofactors for activation of FGFRs [7,24,25].

Though structurally homologous, FGFR family members exhibit different binding specificities to subsets of the 18 FGF ligands [8,10]. While some FGFs, namely FGF1 (acidic FGF) and FGF2 (basic FGF), show binding redundancy among the FGF receptors, others bind to sole members or only some of the family [8]. This variety in ligand-binding specificity is enhanced by tissue-specific alternative splicing of the third Ig-like domain D3 of FGFRs 1–3 [10,11,26,27] and can be attributed, in part, to consequent changes in FGF–FGFR contacts at the βC′-βE region of D3 [13].

The extracellular domain also displays receptor autoinhibition mechanisms, realised by the blocking of FGF binding by domain D1 and the D1–D2 ‘acid-box’ linker [23]; studies of alternative splicing of this region and biophysical analyses suggest that autoinhibition is mediated by competition between ‘acid box’ and heparan sulfate binding, and through back-binding of D1 to the FGF binding-site on domains D2 and D3 [28–31].

## Dimerisation of FGFRs at the transmembrane domain and an alternative stimulation model

The dimerisation of the extracellular domains of FGFRs presumably positions the C-terminal ends of D3 domains such that the intracellular tyrosine kinase domains are arranged to perform *trans*-autophosphorylation; this would require the passing of spatial and conformational information across the plasma membrane. Studies of FGFR transmembrane domains (TMDs) are sparse; however, nuclear magnetic resonance studies of FGFR3 transmembrane domain in d_38_-dodecylphosphocholine/d_29_-sodium dodecyl sulfate (9/1) micelles revealed a symmetric left-handed dimer of helices with 3_10_ and alpha-helical character (Figure 2) [32]. In this structure, the two helices cross one another with an angle of 23°, approximately at the midpoint of the helix length. However, this crossing does not occur at the GxxxG-like motifs of the helices which are observed at dimerisation interfaces of other receptor tyrosine kinases [33–35] and lie immediately upstream of the NMR-observed cross-point. The canonical model of receptor tyrosine kinase activation proceeds via ligand-induced dimerisation, but recent evidence suggests that FGFRs 1–3 may possess intrinsic dimerisation potential even when unliganded [36,37]. Consequently, an allosteric activation model featuring ligand-induced conformational change could be more appropriate for FGFRs (Figure 1), similar to that of insulin receptor tyrosine kinase [38]. Under this model, it is expected that there will be more than one dimerisation state of the transmembrane domain, reflecting different ligand binding at the extracellular domains. The extent of conformational change induced by ligand binding, perhaps manifested as the degree of C-terminal helix separation, could correlate to the level of kinase activation and signalling outcome. Altogether, these suggest that the NMR-observed symmetrical dimer may correspond to the basal dimerisation state of the receptor, while the alternative dimer interface at the GxxxG-like motifs may be that of the fully active state [32]. Additional intermediate activation states with alternative dimerisation interfaces cannot be ruled out. Further independent data lend support to the allosteric model: crystal structures of the extracellular region of FGFR2c bound to FGF8b and FGF2 show variation in the distances between D3 domain C-termini of ∼35 Å and ∼46 Å, respectively [10]. Furthermore, Förster resonance energy transfer (FRET)-based studies of FGF1 and FGF2 binding to FGFRs 1–3 show differences in ligand-induced helix separation, and at least in the case of FGFR3, this further correlates with the level of receptor (auto)phosphorylation [27,36].

## The heart of the action: the FGFR tyrosine kinase domain

The intracellular ‘split’ tyrosine kinase domain of FGFRs shares the prototypical bi-lobed kinase fold (Figure 2) [39–42]. Binding of both adenosine triphosphate (ATP) and substrate occurs at the cleft between the two lobes. Nucleotide binding is facilitated by interactions with N-lobe residues of the hinge region, the glycine-rich P-loop (or nucleotide binding loop) which folds over and encloses ATP for phosphotransfer, and of conserved residue K514 (FGFR1) of helix αC (stabilised by salt-bridge formation with equally conserved E531 (FGFR1)). On the other hand, substrate binding is orchestrated by the C-lobe. Phosphorylation is catalysed by an invariant aspartate residue (D623 in FGFR1) of the His-Arg-Asp (HRD) motif, conserved among protein kinases and located on the C-lobe in the αE-β7 (catalytic) loop. Thus, to attain a catalytically competent state, the N- and C-lobes of the kinase require rotation towards one another during transition from the inactive state. During receptor activation, the kinase domains autophosphorylate one another in the dimer, firstly at Y653 (FGFR1) of the YYKK motif in the activation loop (A-loop) [43]. Seven phosphorylatable tyrosine residues have been identified in FGFR1 (Y463, Y583, Y585, Y653, Y654, Y730 and Y766), five of which are phosphorylated in an ordered fashion *in vitro* [43–46]. Tyrosine residues Y653 and Y654 are essential for kinase activity and their phosphorylation increases catalytic activity 50–100 and 500–1000 fold, respectively [43]. Other phospho-Tyr residues serve as docking sites for SH2 domain-containing adaptor proteins for the stimulation of downstream signalling cascades (Figure 1); for example, phospho-Y766 of FGFR1 serves as a binding-site for phospholipase Cγ (PLCγ) [47,48]. Likewise, Y724 of FGFR3 (equivalent to Y730 of FGFR1) appears to play a central role in FGFR3-mediated signalling, affecting activation of phosphoinositide 3-kinase (PI3K), signal transducer and activator of transcription protein (STAT) and mitogen-activated protein kinase (MAPK) pathways [49,50]. Immediately upstream of the kinase domain, the juxtamembrane domain (JMD) serves as a further site for coupling of receptor activation to downstream signalling cascades; here, the phosphotyrosine-binding domain of FGFR substrate 2 (FRS2α) binds constitutively to FGFR1 in a non-canonical, phosphotyrosine-independent manner and upon its own FGFR-dependent phosphorylation acts as a scaffold for Grb2 adaptor protein for MAPK signalling [51–53].

## Activity regulation of the kinase domain

To *trans*-autophosphorylate in response to ligand binding, the FGFR kinase domain requires an intrinsic basal kinase activity. This requirement has led to a ‘two-state’ dynamic equilibrium model of kinase activation wherein a kinase can exhibit ensembles of a rigid, catalytically ‘inhibited’ state, or a dynamic, conformationally heterogeneous active state [54,55]. Under this model, kinase activity can be fine-tuned by shifting the kinase population between these two states. It is essential that kinases are tightly regulated, ensuring the ability for *trans*-autophosphorylation while preventing overstimulation of signalling cascades; dysregulation of FGFR kinases underpins a plethora of pathologies including developmental abnormalities such as achondroplasia, hypochondroplasia and lacrimo-auriculo-dento-digital (LADD) syndrome, and a host of human cancers [3,56,57].

**Figure 3.**
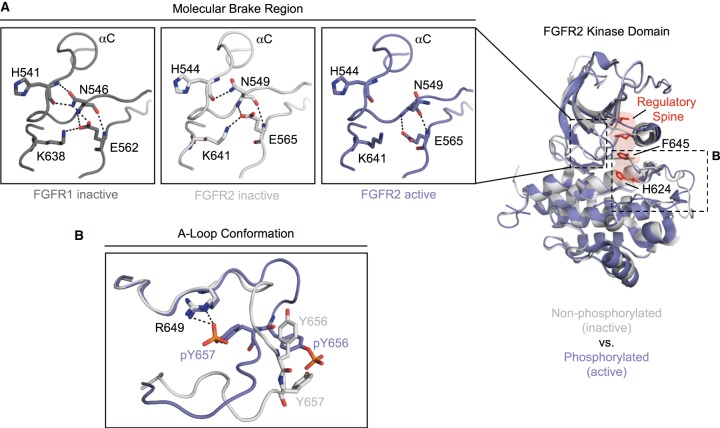
Comparison of active and inactive FGFR kinase domain states. (**A**) Structural overlay (right) of non-phosphorylated, inactive FGFR2 kinase domain (light grey) (PDB: 2PSQ) and phosphorylated, active FGFR2 kinase domain (blue) (PDB: 2PVF), both in cartoon representation. Additionally, in the active kinase domain, the kinase regulatory spine and two participating residues, H624 of the HRD motif and F645 of the DFG motif, are highlighted in red sticks and surface representation. During kinase activation, the molecular brake hydrogen bonding network between H544, N549, E565, and K641 of FGFR2 is broken, as illustrated in the expanded sections (left). The same regions in the inactive state of FGFR1 kinase (PDB: 4V01) (dark grey) with the corresponding H541, N546, E562, and K638 residues are also presented (far left), illustrating the conservation of this feature among FGFRs. (**B**) Structural differences in A-loop conformation in active and inactive FGFR2 kinase domains where phosphorylation-dependent salt bridge interactions between R649 and phospho-Y657 (pY657) stabilise an extended conformation of the loop in the activated kinase.

In the basal, unstimulated state, the kinase domain is autoinhibited and has minimal kinase activity. Mechanisms of autoinhibition vary between kinases [2], and in FGFRs is achieved by the steric blocking of substrate Tyr binding by the protein tyrosine kinase-invariant P663 (FGFR1) at the C-terminal end of the A-loop [39]. Additionally, the so-called molecular brake of FGFR kinases, located at the kinase hinge, has a critical role in establishing autoinhibition and, via its release, activation (Figure 3) [40]. This structural motif is composed of a hydrogen bonding network between FGFR2 residues H544 and N549 (of the αC–β4 loop), E565 (of the kinase hinge) and K641 (of β8) in autoinhibited kinases (H541, N546, E562 and K638, respectively, in FGFR1), but is found to dissociate in activated kinase domain crystal structures (Figure 3) [40]. The release of the molecular brake occurs concomitantly with local A-loop conformational changes from an autoinhibited, substrate-blocked to an extended conformation, and the global conformational rotation of the αC helix (and N-lobe) towards the C-lobe, facilitating the generation of a catalytically competent state. The extended active A-loop conformation of the kinase is stabilised by salt-bridge interactions between phospho-Y657 (of FGFR2, equivalent to Y654 of FGFR1) and conserved residue R649 (of FGFR2), also of the A-loop (Figure 3) [40]. Residues of the molecular brake region are mutated in patients with dwarfism and glioblastoma, highlighting its importance in kinase activity regulation [40,58].

Additional structural features of the kinase domain, including Asp-Phe-Gly (DFG)-motif conformation and kinase hydrophobic spines, are indicators of kinase activity status [59,60]. The DFG-motif, located at the start of the A-loop, classically exists in one of two states: the catalytically competent DFG-in and catalytically incompetent DFG-out state [61–63]. When in the DFG-in state, the Asp residue of the DFG-motif plays an essential role in ATP binding through coordination of all three phosphate groups of ATP, either directly or via magnesium ions; these interactions are not possible in the DFG-out state [62]. Furthermore, the ‘flipping’ of the DFG motif by ∼180° to its DFG-out position breaks the hydrophobic regulatory spine of the kinase. The regulatory spine is a structural entity that spans the N- and C-lobes of a kinase, composed of H624 (FGFR2) of the HRD motif, F645 (FGFR2) of the DFG motif and aliphatic side-chains of residues located on N-lobe elements αC and β4 (Figure 3). Conserved across kinases, its assembly is a hallmark of the active kinase state. A second hydrophobic spine, the catalytic spine, is assembled upon ATP binding, where the adenine base bridges further hydrophobic entities in the N- and C-lobes [59,64]. Recent analysis of inhibited, partially activated and fully activated (phosphorylated) FGFR kinase domains has revealed an interconnected allosteric network at the N- and C-lobe interface, permitting long-distance communication between the molecular brake and A-loop [65]. This allosteric network comprises the molecular brake, an ‘A-loop plug’ element (which holds the loop in a substrate-binding incompatible conformation), and hydrophobic patches of residues termed the DFG-latch and αC-tether. While these are distinct from those elements discussed previously, they are intimately associated with DFG-motif and αC conformational states. Strikingly, naturally found mutations within these elements alter the activity level of the kinase *in vitro*, with combined double mutations in different elements demonstrating additive effects, reflecting a population shift in the two-state dynamic equilibrium [65].

## Dysregulation of FGFRs

**Figure 4.**
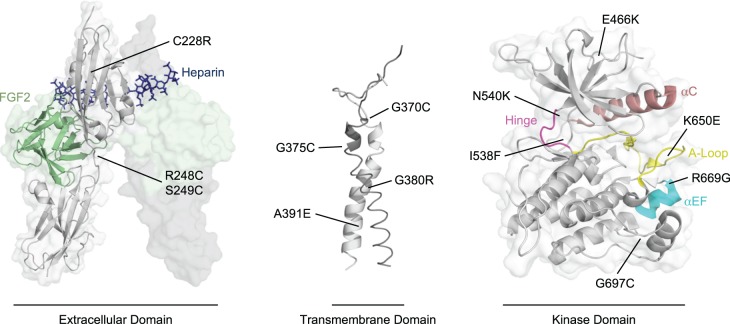
Point mutations of FGFR3. The locations of a selection of developmental disease and cancer-associated point mutations of FGFR3 in the extracellular domain (left) (PDB: 1FQ9), the transmembrane domain (middle) (PDB: 2LZL), and the kinase domain (right) (PDB: 4K33), as discussed in the text. As no FGFR3 ligand-dimerised extracellular domain structure is available, the extracellular domain of FGFR1 in complex with FGF2 is shown, illustrating the localisation of FGFR3 point mutations to regions which could generate similar dimer structures in a ligand-independent manner. Similarly, in the kinase domain, the αC helix (salmon), the αEF helix (cyan), the hinge region (magenta), and the A-loop (yellow) are highlighted to illustrate the localisation of many point mutations to important regulatory elements of the kinase domain.

Subversion of FGFR kinase regulation and receptor hyperactivity is achieved in various ways, from overexpression of FGFRs and/or FGF ligands, to point mutations and gene fusion events [66]. Components of the FGF signalling pathways are the most frequently mutated kinases carrying non-synonymous somatic mutations in human cancers [67]. Though found in all FGFRs, point mutations occur most commonly in FGFR3 and are located in all three receptor domains (Figure 4). Consequently, here we focus on FGFR3 aberrations, though in many instances corresponding mutations can be found in FGFRs 1, 2 and 4 also. One can envisage that point mutations cause ligand-independent activity through either stabilisation of active conformational states or destabilisation of autoinhibitory states.

Point mutations in the extracellular domain of FGFR3, such as R248C/S249C (D2-D3 linker, thanatophoric dysplasia and keratosis [68,69]) and C228R (D2 domain, carcinoma [67,70]) are thought to recapitulate stimulation of the receptor in a ligand-independent manner through obligate receptor dimerisation. This is thought to be achieved by disulfide cross-linking of the extracellular domain and/or by induction of appropriate extracellular domain conformational arrangements for activation of the intracellular kinase domain. To this end, extracellular domain mutations are localised to regions where inter-receptor cross-linking may mimic ligand-bound states (Figure 4). Likewise, mutations in the transmembrane domain are believed to stabilise the active (or destabilise the basal) dimerisation interfaces of the helices, shifting the equilibrium towards a ligand-stimulated dimer arrangement. Indeed, cysteine-introducing point mutations such as G375C/G370C (N-terminal end of the transmembrane domain, achondroplasia and keratosis [69,71]) may act to cross-link the dimer at the extracellular domain/transmembrane domain interface and cause separation of the C-terminal ends of the transmembrane helices. Alternatively, substitutions of large, polar residues such as G380R (achondroplasia and hypochondroplasia [72,73]) and A391E (Crouzon syndrome with acanthosis nigricans [74]) in and around the transmembrane dimer interface may destabilise the basal dimerisation state by steric and Coulombic repulsion, or potentially stabilise the active state through hydrogen-bonding [32]. Supportively, energetic dimer stabilisation is observed following A391E substitution in the transmembrane domain [70].

The kinase domain is abundant in point mutations which are typically localised to regulatory elements such as the molecular brake, the A-loop, the kinase hinge, the DFG-latch and others. Recently, an extensive study on the prevalence and activating effect of point mutations in FGFR3 identified N540 (molecular brake) and K650 (A-loop) as mutational hotspots in FGFRs; these also elicit significant stimulation of FGFR3 kinase domain autophosphorylation [75]. Both mutation sites have been extensively characterised; molecular brake mutations are thought to overcome autoinhibitory mechanisms and facilitate transition to the active kinase state [40], while K650E substitution mimics A-loop Y648 (FGFR3) phosphorylation and stabilises the active, extended A-loop conformation [41]. Surprisingly, the study showed that clinical prevalence does not directly correlate with stimulatory effect. For example, R669G, the most activating mutation with respect to FGFR3 kinase domain autophosphorylation, is not a mutation hotspot, whereas G697C (of oral squamous cell carcinomas [76]), an identified FGFR3 mutation hotspot, has no stimulatory effect on kinase activity over wild-type kinase domain under the analysed conditions [75]. R669, due to its location at the C-terminal end of αEF in the C-lobe likely influences kinase activity by effect on the kinase A-loop (Figure 4); in fact, crystallographic evidence suggests that the corresponding residue R675 in FGFR1 contacts residues of the activation loop, stabilising the inactive kinase state. Upon R675 mutation of FGFR1, these contacts are lost and the kinase A-loop instead occupies an ‘open’ active conformation [75]. On the other hand, G697 is located within the αF-αG loop at the base of the C-lobe and does not appear to have any direct interactions with kinase regulatory elements (Figure 4). While the general frequency of G697C substitution in FGFR3 is disputed [77], the lack of stimulatory action by cancer-associated mutations is not unique to G697C; in truth, there are multiple mutations which are neutral-to-destabilising with respect to kinase activity, do not increase kinase domain autophosphorylation nor substrate phosphorylation, yet are nonetheless observed in tumours [75]. Furthermore, numerous deleterious point mutations in the FGFR kinase domain have been identified [75,78]. The role of deleterious, inhibitory and neutral mutations of FGFRs in pathologies is unclear but may be dependent on cellular context, and their purpose may become clearer when evaluated macroscopically with interaction partners and signalling networks in the cell [3]. For example, the destabilising and inactivating point mutations E466K and I538F of FGFR3, like kinase-activating N540K, enhance kinase domain binding to heat shock protein 90 (Hsp90) co-chaperone Cdc37 [79]. As Hsp90 has been implicated in the regulation and activation mechanisms of kinases [80], altered association with cellular chaperones is consistent with the notion that dysregulation of FGFRs by such mutations may play out at the level of the wider interactome of the kinase.

Although of relatively low clinical incidence, oncogenic gene fusions of FGFRs have recently come to light in a variety of cancer types [81,82]. Typically, these fuse self-associating elements of a second protein in frame with the C-terminal end (and less frequently the N-terminal end) of the receptor. Though supporting structural evidence is lacking to date, FGFR gene fusions are expected to cause ligand-independent constitutive kinase activity through fusion protein-induced dimerisation of the receptors, similar to that observed for the TPR-MET kinase fusion [83]. C-terminal fusions also lack exon 19 of the receptor, resulting in the inability to activate PLCγ signalling through loss of its phospho-Tyr binding-site [84]. While a variety of fusion partner genes have been identified for FGFR2 [66], gene fusions of FGFR3 almost exclusively occur with transforming acidic coiled-coil-containing protein 3 (TACC3) [66,84]. These fusions and the FGFR3-BAIAP2L1 (brain-specific angiogenesis inhibitor 1-associated protein 2-like protein 1) fusion are exquisitely sensitive to FGFR-selective inhibitors in urothelial cells, indicating that these aberrations are highly targetable [82,84].

## Progress towards therapies for FGFR-driven diseases

**Figure 5.**
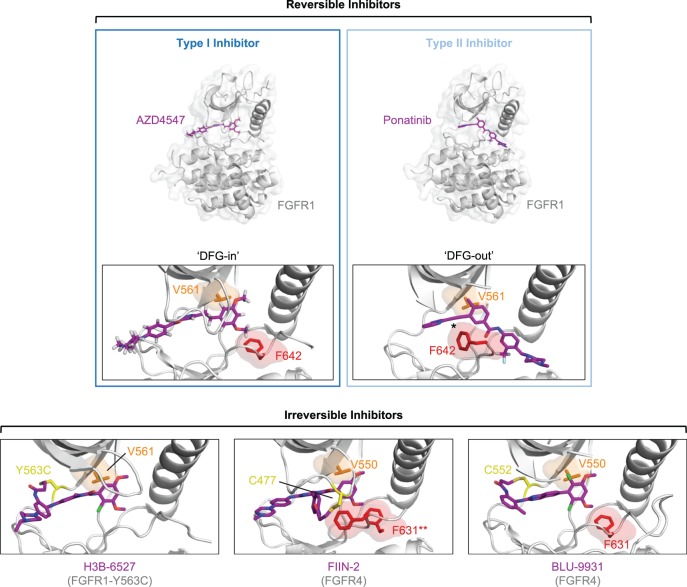
Binding modes of FGFR inhibitors. Crystal structures of inhibitor-bound FGFR1 and FGFR4 kinase domains, illustrating the binding modes of reversible and irreversible (covalent) inhibitors. Reversible inhibitors can be classified into type I and type II inhibitors, differing in their binding modes (top). Type I inhibitors such as AZD4547 bind to active, DFG in-state kinases, whereas type II inhibitors such as ponatinib bind to inactive, DFG out-state kinases. In each instance, FGFR1 kinase domains are shown in full in cartoon representation with transparent surfaces (light grey) and inhibitors in stick representation (purple). Additionally, the inhibitor-binding site is expanded for each with FGFR1 (light grey) in cartoon representation alone, and F642 of the DFG motif (red) and the gatekeeper residue V550 (orange) shown in stick representation with transparent surfaces. In the ponatinib-bound structure, the asterisk (*) indicates the location of the ethynyl group attributed to the ability of ponatinib to accommodate gatekeeper residue mutations and to the multikinase selectivity profile of the inhibitor. The binding modes of three irreversible, covalent inhibitors to FGFR4 and FGFR4 surrogate kinase domain (FGFR1–Y563C) are presented in expanded panels in a similar manner (bottom). In these, where resolved in the crystal structures, the gatekeeper residue and Phe of the DFG motif is shown as above, and the Cys residues utilised in ligand conjugation are highlighted also (yellow). In the FIIN-2-bound structure, F631 (DFG motif, FGFR4) is observed in both the DFG in- and DFG out-states, marked with a double asterisk (**). The structures presented are: FGFR1 kinase domain bound to AZD4547 (PDB: 4V05) and ponatinib (PDB: 4V01); FGFR4 kinase domain bound to BLU-9931 (PDB: 4XCU) and FIIN-2 (PDB: 4QQC) and of FGFR4 surrogate kinase domain (FGFR1 harbouring a Y563C substitution) bound to H3B-6527 (PDB: 5VND).

The finding that aberrant FGFR signalling has driving roles in a plethora of cancers has spurred research interests in the development of anti-FGFR treatments, predominantly taking the form of small molecule kinase inhibitors (Figure 5). FGFR-targeting treatments under clinical development have been extensively reviewed previously [85–87] and will only be summarised here.

Small molecule inhibitors of FGFRs can be classified into non-selective multi-kinase inhibitors and selective FGFR inhibitors. The first efforts to treat FGFR aberrations have made use of non-selective multi-kinase inhibitors such as dovitinib, ponatinib and lucitanib which show pan-FGFR inhibition with nanomolar IC_50_ values against FGFR family members (Table 1). With the notable exception of ponatinib, these compounds are type I inhibitors which bind to the active, DFG-in state of FGFR kinases in an ATP-competitive manner. Often, vascular endothelial growth factor receptors (VEGFRs) and platelet-derived growth factor receptors (PDGFRs) are also targeted by these non-selective FGFR inhibitors. While the ability to target multiple kinases with a single compound may be clinically beneficial under certain circumstances, non-selective multi-kinase FGFR inhibitors typically exhibit lower affinity binding to FGFRs than other targets. Consequently, the use of these non-selective inhibitors as FGFR-targeted therapies is associated with off-target-related toxicities [86]. The broad specificity of many kinase inhibitors has been attributed to the high degree of structural conservation between kinases rendering the development of selective inhibitors challenging, particularly against those in the active state. A second subclass of reversible kinase inhibitors, type II inhibitors, bind to kinases in a DFG-out, inactive state. To generate this kinase state, the Phe residue of the DFG-motif ‘flips’ outwards, breaking the regulatory spine and providing access to an additional hydrophobic pocket from the ATP binding-site (Figure 5) [63]. Type II inhibitors have proved to be generally more selective than their type I counterparts, while also exhibiting considerably slower dissociation kinetics [88,89]; however, the ability for a type II inhibitor to bind to its target is dependent on the propensity of the kinase to ‘visit’ the DFG-out state through conformational sampling, implying that some kinase classes may be innately more amenable to type II inhibition than others. Furthermore, a survey of many kinase inhibitors has established that not all type II inhibitors are necessarily more selective [90]. This is the case for the multi-kinase type II inhibitor ponatinib which was originally developed to target BCR-ABL aberrations harbouring the T315I ‘gatekeeper’ mutation in the ATP binding-site, conferring resistance to earlier generation BCR-ABL inhibitors [91]. While ponatinib is able to accommodate the Thr to Ile mutation of the gatekeeper residue in BCR-ABL through productive interactions with the unsaturated ethynyl bond of the inhibitor, this feature is also likely to be responsible for the relatively poor kinase selectivity of the inhibitor, which additionally exhibits potent pan-FGFR inhibition [91].

**Table 1:** Inhibitors of FGFR

Inhibitor name	Company	Measured IC_50_ (nM, *in vitro*)	Progress in clinical trials (with identifiers)			Ref.
**Multi-kinase inhibitors**							
Ponatinib (AP24534)	ARIAD Pharmaceuticals	FGFR1: 2.2 FGFR2: 1.6 FGFR3: 18.2 FGFR4: 7.7	***Phase II*** NCT02272998	NCT02265341		[91]
Dovitinib (CHIR258, TKI258)	Novartis	FGFR1: 8 FGFR3: 9	***Phase II*** NCT01732107^†^ NCT01676714 NCT01379534	NCT01576380 NCT00790426 NCT01719549	NCT01058434 NCT01831726 NCT00958971	[92]
Lucitanib (E-3810)	Clovis Oncology	FGFR1: 17.5 FGFR2: 82.5 FGFR3: 237.5 FGFR4: >1000	***Phase I*** NCT03117101 ***Phase I/II*** NCT01283945 ***Phase II*** NCT02202746	NCT02109016	NCT02053636	[93]
Nintedanib (BIBF 1120)	Boehringer Ingelheim	FGFR1: 69 FGFR2: 37 FGFR3: 108 FGFR4: 610	***Phase II*** NCT01948141			[94]
ARQ 087 (Derazantinib)	ArQule	FGFR1: 4.5 FGFR2: 1.8 FGFR3: 4.5 FGFR4: 34	***Phase I/II*** NCT01752920 ***Phase II*** NCT03230318			[95]
**FGFR-selective inhibitors**							
AZD4547	AstraZeneca	FGFR1: 0.2 FGFR2: 2.5 FGFR3: 1.8 FGFR4: 165	***Phase I*** NCT01213160 ***Phase I/II*** NCT01824901^‡^ NCT02824133^§^ ***Phase II/III*** NCT02965378^‡^	NCT00979134 NCT01202591^‡^	NCT01791985^‡^	[96]
LYS2874455	Eli Lilly	FGFR1: 2.8 FGFR2: 2.6 FGFR3: 6.4 FGFR4: 6	***Phase I*** NCT01212107	NCT03125239^‡^		[97]
(NVP-)BGJ398 (Infigratinib)	Novartis	FGFR1: 0.9 FGFR2: 1.4 FGFR3: 1 FGFR3^K650E^: 4.9 FGFR4: 60	***Phase I*** NCT01697605 ***Phase II*** NCT02706691 NCT03510455	NCT01004224 NCT02150967	NCT01928459^‡^ NCT01975701	[98]
Debio-1347 (CH5183284)	Debiopharm International	FGFR1: 9.3 FGFR2: 7.6 FGFR3: 22 FGFR4: 290	***Phase I*** NCT01948297 ***Phase I/II*** NCT03344536^‡^			[99]
Erdafitinib (JNJ-42756493)	Janssen	FGFR1: 1.2 FGFR2: 2.5 FGFR3: 3.0 FGFR4: 5.7	***Phase I*** NCT02421185 NCT03238196^‡^ ***Phase I/II*** NCT03473743^‡^ ***Phase II*** NCT03210714 NCT02952573^‡^ ***Phase III*** NCT03390504^‡^	NCT01962532 NCT02699606	NCT01703481 NCT02365597	[100]
INCB054828	Incyte Corporation	FGFR1: 3–50^*^	***Phase I*** NCT03235570 ***Phase I/II*** NCT02393248^‡^ ***Phase II*** NCT03011372	NCT02872714	NCT02924376	[101]
Rogaratinib (BAY1163877)	Bayer	FGFR1: 12–15 FGFR2: <1 FGFR3: 19 FGFR4: 33	***Phase I*** NCT01976741 ***Phase I/II*** NCT03473756^‡^ ***Phase II/III*** NCT03410693			[102]
(NVP)FGF401	Novartis	FGFR4: 1.1	***Phase I/II*** NCT02325739^‡^			[103]
PD173074	Pfizer	FGFR1: 22–25 FGFR3: 29	N/A			[104,105]
PD166866	Pfizer	FGFR1: 52.4	N/A			[106]
SSR128129E	N/A	FGFR1: 1900	N/A			[107]

A selection of small molecule multi-kinase and FGFR-selective reversible inhibitors, their measured *in vitro* IC_50_ values and clinical trial status. Key: ‘Ref.’, reference.

*IC_50_ value measured using in cell assays.

† Trial terminated due to funding.

‡ Drug combination study.

§ Trial suspended.

To address the toxicity issues of multi-kinase inhibitors, efforts have been made to develop FGFR-selective kinase inhibitors, yielding numerous reversible type I inhibitor compounds with FGFR1–3 and pan-FGFR activities (Table 1). Of these, AZD4547, a potent inhibitor of FGFRs 1–3, has shown promising responses in preclinical and phase I clinical trials, particularly towards tumours with FGFR amplifications [108–110]. Several phase II clinical trials evaluating the efficacy of AZD4547 alone or in combination with other compounds are active or have completed. However, preclinical studies have also indicated that resistance can be conferred to AZD4547 via the gatekeeper mutation V555M in FGFR3 [111], much like that in BCR-ABL, highlighting the need for continued inhibitor development and the personalisation of FGFR-targeted therapies in the clinic. Towards this end, a second-generation FGFR-selective inhibitor Debio-1347 has been developed which has a different chemical scaffold to AZD4547, PD173074 and BGJ398, and has shown inhibition efficacy against Ba/F3 cells harbouring a FGFR2 fusion with V564F gatekeeper mutation [99]. Despite efforts, no FGFR-selective type II inhibitors in the vein of ponatinib have yet been reported, though several irreversible, covalent inhibitors of FGFRs have been developed. Unlike reversible inhibitors, covalent inhibition confers the advantage of partially circumventing high *in vivo* ATP concentrations [112]. Furthermore, covalent inhibition has facilitated the development of isoform-selective inhibitors; many these covalent inhibitors are highly selective for FGFR4 (Table 2). This FGFR isoform selectivity has been achieved in at least three cases (H3B-6527, BLU-9931 and BLU-554) through the use of the FGFR4-unique C552 residue of the hinge region which is occupied by a Tyr residue in the corresponding position in FGFRs 1–3 (Figure 5) [113–115]. Conversely, pan-FGFR covalent inhibition has been achieved through use of the FGFR-conserved C477 residue (FGFR4) in the cases of inhibitors FIIN-2 and FIIN-3 (Table 2), both of which also exhibit activities against FGFR2 harbouring gatekeeper mutations in cell-based assays [116]. Intriguingly, a crystal structure of FIIN-2 bound to FGFR4 indicates that the inhibitor can bind to both DFG-in and DFG-out states of the kinase, though the inhibitor does not occupy the additional hydrophobic pocket which is accessible in the DFG-out state (Figure 5). The significance, if any, of being able to bind to both states is unclear; however, FIIN-2 could form the foundation for development of next-generation type II-like covalent inhibitors. At the time of writing, four covalent FGFR inhibitors (PRN1371, TAS-120, H3B-6527 and BLU-554) are recruiting for phase I clinical trials.

**Table 2:** Irreversible, covalent FGFR-selective inhibitors under development

Inhibitor name	Company	Measured IC_50_ (nM, *in vitro*)	Progress in clinical trials (with identifiers)	Ref.
PRN1371	Principia Biopharma	FGFR1: 0.6 FGFR2: 1.3 FGFR3: 4.1 FGFR4: 19.3	***Phase I*** NCT02608125	[112]
TAS-120	Taiho Oncology	FGFR1: 3.9 FGFR2: 1.3 FGFR3: 1.6 FGFR4: 8.3	***Phase I/II*** NCT02052778	[117]
BLU-554	Blueprint Medicines Corporation	FGFR1: 624 FGFR2: 1202 FGFR3: 2203 FGFR4: 5	***Phase I*** NCT02508467	[118,119]
BLU-9931	Blueprint Medicines Corporation	FGFR1: 591 FGFR2: 493 FGFR3: 150 FGFR4: 3	N/A	[115]
FIIN-2	N/A	FGFR1: 3.09 FGFR2: 4.3 FGFR3: 27 FGFR4: 45.3	N/A	[116]
FIIN-3	N/A	FGFR1: 13.1 FGFR2: 21 FGFR3: 31.4 FGFR4: 35.3	N/A	[116]
H3B-6527	H3 Biomedicine, Eisai Incorporation	FGFR1: 320 FGFR2: 1290 FGFR3: 1060 FGFR4: <1.2	***Phase I*** NCT02834780	[114]

FGFR-selective inhibitors that have an irreversible, covalent mode of action, their measured *in vitro* IC_50_ values and their clinical trial status. Key: ‘Ref.’, references.

FGFR-targeted therapies are not limited to the tyrosine kinase domain only; there have been multiple efforts to target the extracellular domains of FGFRs also, offering further opportunities for isoform-selective inhibition (Table 3). This is best exemplified by the development of anti-FGFR2 and anti-FGFR3 monoclonal antibodies/antibody-drug conjugates [120–124]. FP-1039, an FGF-ligand trap composed of an FGFR1 extracellular domain-IgG1 fusion which is able to inhibit tumour growth in xenograft models, has also been developed [125]. Furthermore, a novel small molecule inhibitor (SSR128129E) which binds to FGFR extracellular domains in a non-FGF competitive manner but induces selective, allosteric inhibition of receptor internalisation and ERK1/2 signalling has been described [107,126]. Lastly, there has also been exploration of the use of antisense therapy for targeting FGFR4 in obesity patients (Table 3) [127].

While improved selectivity of therapies may be beneficial to target specific FGFR aberrations, it is important to recognise that clinical efficacy and selectivity are not necessarily related. Highly selective FGFR-targeted therapies may also be more prone to resistance development if not used in combination strategies. For example, in addition to the gain of gatekeeper mutations detailed above, resistance to FGFR inhibitors has also been acquired in cell lines harbouring FGFR3 amplification by switching to ErbB family signalling [128]. Moreover, while one aim of FGFR-selective inhibitor development is to overcome off-target effects of multi-kinase inhibitors, FGFR-selective therapies are not immune to side effects, exemplified by toxicity profiles associated with FGFR-selective inhibitors in the clinic [86]. FGFRs are still a relatively novel target and many anti-FGFR programmes are still in their early stages; however, with multiple clinical trials active or recruiting, in many cases with participants with specific FGFR aberrations, we should soon glean further insights that help to improve our approaches to treatment of FGFR dysregulation.

**Table 3:** Alternative therapies for FGFR aberrations under development

Molecule name	Company	Target	Progress in clinical trials (with identifiers)		Ref.
**FGF ligand traps**					
FP-1039 (GSK3052230)	Five Prime Therapeutics	FGF2 and others	***Phase II*** NCT01244438*		[125]
**Anti-FGFR monoclonal antibodies**					
Bemarituzumab (FPA144)	Five Prime Therapeutics	FGFR2b	***Phase I*** NCT02318329	NCT03343301^†^	[120]
BAY1179470	Bayer	FGFR2	***Phase I*** NCT01881217		[122]
LY3076226	Eli Lilly	FGFR3	***Phase I*** NCT02529553		[123]
MFGR1877S	Genentech	FGFR3	***Phase I*** NCT01122875	NCT01363024	[124]
**Antisense therapy**					
ISIS-FGFR4RX	ISIS Pharmaceuticals	FGFR4	***Phase II*** NCT02476019		[127]

Additional non-kinase-domain inhibitor-based therapies under development, their targets and their clinical trial status. Key: ‘Ref.’, references.

*Trial withdrawn.

† Drug combination study.

## Conclusions and perspectives

Since their first description, it has been established that FGFRs play crucial roles in a host of physiological processes which when dysregulated result in a plethora of pathologies. From an extensive range of studies covering FGFR expression, structure and function, among others, mechanisms of FGFR regulation and activation have come to light, and good progress has been made in the development of anti-FGFR therapies. Despite this, due to the complexity of FGFR signalling inputs, outputs and FGFR interactomes, and difficulties faced with the biochemical and biophysical characterisation of full-length receptors, we are still far from an integrated understanding of FGFR biology. Crucially, mechanisms of activation in the context of the full-length receptor are unclear and will remain unresolved until structures of full-length FGFRs in autoinhibited, ligand-activated and pathogenically activated modes are solved. In fact, to date there are no high-resolution full-length structures of any receptor tyrosine kinase, severely limiting our understanding of this highly important class of kinases. Equally, while it is recognised that activation of FGFRs can lead to differential activation of intracellular signalling cascades, the underlying molecular basis of how this occurs and of how cellular context influences phenotypic outcome remain poorly understood. We anticipate that advances will be made in addressing these and other remaining questions in the coming decades, and with this, new and improved strategies for treatment of disorders arising due to aberrant FGFR signalling will develop.

## Abbreviations

ATP: adenosine triphosphate
BAIAP2L1: brain-specific angiogenesis inhibitor 1-associated protein 2-like protein 1
D1–3: immunoglobulin (Ig)-like domains 1–3
DFG-motif: Asp-Phe-Gly motif
ECD: extracellular domain
ERK1/2: extracellular signal-regulated kinases 1/2
FGF: fibroblast growth factor
FGFR: fibroblast growth factor receptor
FRET: Förster resonance energy transfer
FRS2α: FGFR substrate 2
Grb2: growth factor receptor-bound protein 2
HRD motif: His-Arg-Asp motif
Hsp90: heat shock protein 90
JMD: juxtamembrane domain
KD: kinase domain
LADD syndrome: lacrimo-auriculo-dento-digital syndrome
MAPK: mitogen-activated protein kinase
PDGFR: platelet-derived growth factor receptor
PI3K: phosphoinositide 3-kinase
PLCγ: phospholipase Cγ
P-loop: nucleotide binding loop
RTK: receptor tyrosine kinase
SH2 domain: Src-homology 2 domain
STAT: signal transducer and activator of transcription protein
TACC3: transforming acidic coiled-coil-containing protein 3
TMD: transmembrane domain
VEGFR: vascular endothelial growth factor receptor
